# Longitudinal trends in lipid profiles during pregnancy: Association with gestational diabetes mellitus and longitudinal trends in insulin indices

**DOI:** 10.3389/fendo.2022.1080633

**Published:** 2023-01-13

**Authors:** Lixia Shen, Dongyu Wang, Yihong Huang, Lisha Ye, Caixia Zhu, Shaofeng Zhang, Shiqin Cai, Zilian Wang, Haitian Chen

**Affiliations:** Department of Obstetrics and Gynecology, The First Affiliated Hospital of Sun Yat-sen University, Guangzhou, China

**Keywords:** lipid profiles, insulin, gestational diabetes mellitus, insulin resistance, pregnancy, trends

## Abstract

**Objective:**

To investigate the correlation of trends in lipid profiles from first to second trimester with trends in insulin indices and gestational diabetes mellitus (GDM).

**Methods:**

Secondary analysis of an ongoing prospective cohort study was conducted on 1234 pregnant women in a single center. Lipid profiles, glucose metabolism and insulin indices were collected in the first and second trimesters. Trends in lipid profiles were divided into four subgroups: low-to-low, high-to-high, high-to-low and low-to-high group. Insulin indices including homeostasis model assessment of insulin resistance and quantitative insulin sensitivity check index were calculated to evaluate insulin resistance (IR). Trends in insulin indices were described as: no IR, persistent IR, first-trimester IR alone and second-trimester IR alone. Pearson correlation analysis and multivariate logistic regression were performed to assess the associations of lipid profiles subgroups with insulin indices and GDM.

**Results:**

First- and second-trimester total cholesterol (TC), triglycerides (TG) and high-density lipoprotein cholesterol were strongly correlated to first- and second-trimester insulin indices. Only TG had a sustained correlation with glucose metabolism indices. High-to-high low-density lipoprotein cholesterol (LDL-c) was an independent risk factor for GDM. High-to-high TG and high-to-low TG groups were independent risk factors for persistent IR. High-to-high TG and low-to-high TG groups were independent risk factors for second-trimester IR alone.

**Conclusion:**

TG has a sustained correlation with insulin indices and glucose metabolism indices. Persistently high TG is an independent risk factor for persistent IR and second-trimester IR alone. Regardless of whether pregnant women have first-trimester IR, lower TG levels help reduce the risk for persistent IR or subsequent development of IR. These results highlight the benefit of lowering TG levels in early and middle pregnancy to prevent the development of IR.

## Introduction

Gestational diabetes mellitus (GDM) is one of the most common complications during pregnancy in China (1–3) and is associated with short-term and long-term adverse outcomes in the mother and her offspring. GDM increases the risks of maternal and perinatal morbidities, such as cesarean delivery, preterm labor, fetal macrosomia, neonatal hypoglycemia and the need for neonatal unit admission (4, 5). Moreover, women with a previous history of GDM and the offspring of affected pregnancy are predisposed to future type 2 diabetes mellitus, obesity, and cardiovascular and metabolic diseases (6–8).

Excessive insulin resistance (IR), commonly calculated by two insulin indices: the homeostasis model assessment of insulin resistance (HOMA-IR) and quantitative insulin sensitivity check index (QUICKI), is a contributory factor in the pathogenesis of GDM. It is suggested that IR during pregnancy increases the risk of GDM (9–11). Furthermore, GDM with IR increases adverse pregnancy outcomes, such as preterm labor, large for gestational age (LGA) fetuses and hypertensive disorders of pregnancy, compared with GDM without IR (12, 13).

Dyslipidemia plays important roles in impaired glucose metabolism and IR (14, 15). These three conditions interact and can aggravate adverse maternal and fetal outcomes. Previous studies have shown that dyslipidemia during pregnancy is associated with the subsequent development of GDM, IR, preeclampsia and LGA (12, 13, 16, 17). However, it is unclear whether the longitudinal trends in IR status parallel trends in lipid profiles throughout pregnancy. We hypothesized that trends in lipid profiles from first to second trimester would influence the incidence of GDM and relate to the trends in insulin indices.

This study aimed to investigate the correlation of trends in lipid profiles from first to second trimester with trends in insulin indices and GDM.

## Material and methods

This secondary analysis of data from a prospective cohort study included pregnant women who performed routine prenatal care at First Affiliated Hospital of Sun Yat-sen University between July 2021 and July 2022.

Inclusion criteria for the study were: age ≥18 years old; singleton pregnancy; underwent blood tests for lipid profiles and fasting insulin in both the first (<14 weeks of gestation) and second (24-28 weeks of gestation) trimesters; Exclusion criteria were: women with pregestational diabetes mellitus or impaired fasting glucose; pregnancy loss or termination of pregnancy before 28 weeks of gestation; missing data on lipid profiles, insulin or GDM diagnosis.

All the following data were extracted from the electronic medical records in our hospital: maternal demographic characteristics including maternal age, body mass index (BMI, calculated in kg/m^2^) before pregnancy, method of conception, smoking, first-degree family history of diabetes mellitus and parity; blood test results in the first and second trimesters including lipid profiles: total cholesterol (TC), triglycerides (TG), high-density lipoprotein cholesterol (HDL-c) and low-density lipoprotein cholesterol (LDL-c); glucose metabolism indices: hemoglobin A1c (HbA1c), oral glucose tolerance test (OGTT) result between 24 and 28 weeks of gestation, first-trimester fasting plasma glucose (FPG, second-trimester FPG was included in OGTT test); and insulin indices: fasting insulin (FINS), HOMA-IR and QUICKI.

Pregestational diabetes mellitus included diabetes diagnosed before pregnancy and overt diabetes in pregnancy (18). Impaired fasting glucose was defined as FPG 5.6-6.9 mmol/L in the first trimester (19). GDM was diagnosed when one or more of the following one-step 75g OGTT criteria were first met between 24 and 28 weeks of gestation: FPG 5.1-6.9 mmol/L, 1-hour plasma glucose ≥10.0 mmol/L or 2-hour plasma glucose 8.5-11.0 mmol/L (20).

### Definition and subgroups of abnormal lipid profiles and insulin indices

Each lipid parameter was stratified into two levels. For TC, TG and LDL-c, concentrations above the 75th percentile of the study population were defined as high, otherwise were specified as low. For HDL-c, concentrations below the 25th percentile of the study population were defined as low, otherwise were specified as high. Accordingly, four lipid subgroups (G1 to G4) were derived based on the lipid levels from first to second trimester. Among them G1 denoted low-to-low group, G2 denoted high-to-high group, G3 denoted high-to-low group, and G4 denoted low-to-high group, respectively. For example, for TG, women in G1 group represented those whose TG levels were below the 75th percentile of the study population across the two trimesters.

Insulin indices including HOMA-IR and QUICKI were applied to evaluate IR. HOMA-IR was calculated as FINS (μU/mL) × FPG (mmol/L)/22.5. QUICKI was calculated as 1/(Log FPG (mg/dL) + Log FINS (μU/mL). In the present study, IR was defined by a HOMA-IR index above the 75th percentile of the study population, and a QUICKI index lower than the 25th percentile of the study population (21). Four HOMA-IR subgroups were derived: IR-H1 group, defined as pregnancies without high HOMA-IR in both the first and second trimesters; IR-H2 group, defined as pregnancies with persistently high HOMA-IR in both the first and second trimesters; IR-H3 group, defined as pregnancies with first-trimester high HOMA-IR alone; and IR-H4 group, defined as pregnancies with second-trimester high HOMA-IR alone. Similarly, four QUICKI subgroups were derived: IR-Q1 group, defined as pregnancies with persistently high QUICKI in both the first and second trimesters; IR-Q2 group, defined as pregnancies with persistently low QUICKI in both the first and second trimesters; IR-Q3 group, defined as pregnancies with first-trimester low QUICKI alone; and IR-Q4 group, defined as pregnancies with second-trimester low QUICKI alone. Based on that, trends in IR were described as no IR, persistent IR, first-trimester IR alone and second-trimester IR alone.

### Statistical analysis

Continuous variables were presented in mean ± standard deviation and categorical variables were represented in counts and proportions. Continuous variables were compared across different subgroups by Mann-Whitney U test, and categorical variables were compared by χ2 test or continuity correction test. Pearson correlation analysis was performed to evaluate the associations among lipid concentrations, insulin indices and glucose metabolism indices.

Multivariate logistic regression was performed to explore whether trends in lipid profiles from first to second trimester were associated with trends in insulin indices and GDM. The adjusted odds ratios (aOR) and 95% confidence intervals (CI) were calculated by adjusting maternal age, BMI before pregnancy, conception by *in vitro* fertilization (IVF), first-degree family history of diabetes mellitus, smoking and multiparous. The relationships of lipid profiles with insulin indices and GDM were also explored within subgroups stratifying BMI before pregnancy, which were categorized as underweight (< 18.5 kg/m^2^), normal weight (18.5-23.9 kg/m^2^), overweight or obese (≥ 24.0 kg/m^2^).

Statistical software package SPSS Statistics 26.0 (SPSS Inc., Chicago, IL, USA) and R software (version 4.1.3) were used for data analyses. In all analyses, *P* value of less than 0.05 was considered statistically significant.

## Results

### Baseline characteristics, lipid profiles, glucose metabolism indices and insulin indices in the study population

The total study population consisted of 2021 women with singleton pregnancies that underwent all the required blood tests during the study period. Seven hundred and eighty-seven cases were excluded due to pregnancy loss or termination of pregnancy (n=34), preexisting diabetes mellitus (n=55) and missing data (n=698). The remaining 1234 cases comprised 233 (18.9%) GDM and 1001 (81.1%) non-GDM cases.

In the present study, the 75th percentile of HOMA-IR in the study population were 1.60 and 1.96 in the first and second trimesters, respectively; and the 25th percentile of QUICKI were 0.36 and 0.34 in the first and second trimesters, respectively. Accordingly, 778 (63.0%) women had persistently low HOMA-IR in both the first and second trimesters (IR-H1). By contrast, 159 (12.9%), 148 (12.0%) and 149 (12.1%) women had high HOMA-IR in both trimesters (IR-H2), in first trimester alone (IR-H3) and second trimester alone (IR-H4), respectively. Similarly, 783 (63.4%), 155 (12.6%), 142 (11.5%) and 154 (12.5%) women had persistently high QUICKI (IR-Q1), persistently low QUICKI (IR-Q2), first-trimester low QUICKI alone (IR-Q3) and second-trimester low QUICKI alone (IR-Q4), respectively.

In the first and second trimesters, high TC, TG and LDL-c levels were defined as above 5.50 and 7.00 mmol/L, 1.59 and 2.49 mmol/L, 3.10 and 4.03 mmol/L, respectively; low HDL-c level was defined as below 1.56 and 1.83 mmol/L, respectively in the present study. Each lipid parameter was divided into four subgroups (G1-G4) as described in the methods part. Over half of our study population had normal lipid levels in the first and second trimesters (numbers and percentages in each lipid were TC: 858, 69.5%; TG: 813, 65.9%; HDL-c: 825, 66.9% and LDL-c: 817, 66.2%, respectively). Conversely, numbers and percentages in women with persistently abnormal lipid levels were TC: 175, 14.2%; TG: 194, 15.7%; HDL-c: 193, 15.6% and LDL-c: 194, 15.7%, respectively.

Compared to non-GDM group, maternal age, BMI before pregnancy, the proportion of conception by IVF, first-degree family history of diabetes mellitus and first-trimester TC and LDL-c were higher in GDM group (**Table 1**). TG, HbA1c, FINS and HOMA-IR were higher, and QUICKI was lower in GDM group than those in non-GDM group in both first and second trimesters (**Table 1**).

**Table 1:** Maternal characteristics, lipid profiles and glucose metabolism and insulin indices in the first and second trimesters in women with and without gestational diabetes mellitus.

Characteristics	GDM N=233	Non-GDM N=1001	*Z* or χ2 value	*P* value
Maternal age, years	33.30 ± 4.60	31.14 ± 4.12	6.581	< 0.001
BMI, kg/m^2^	21.94 ± 3.05	20.99 ± 2.71	4.339	< 0.001
Conception by IVF, n (%)	60 (25.8)	161 (16.1)	12.014	0.001
Smoking, n (%)	2 (0.9)	10 (1.0)	0.039	1.000
Family history of diabetes mellitus, n (%)	22 (9.4)	44 (4.4)	9.508	0.002
Multiparous, n (%)	89 (38.2)	323 (32.3)	2.988	0.084
Blood tests in the first trimester				
TC, mmol/L	5.11 ± 0.97	4.94 ± 0.79	2.466	0.014
TG, mmol/L	1.47 ± 0.56	1.33 ± 0.53	4.015	< 0.001
HDL-c, mmol/L	1.78 ± 0.32	1.79 ± 0.33	-0.076	0.939
LDL-c, mmol/L	2.88 ± 0.68	2.75 ± 0.55	2.876	0.004
HbAlc, %	5.25 ± 0.34	5.11 ± 0.29	5.670	< 0.001
FPG, mmol/L	4.42 ± 0.37	4.32 ± 0.33	4.168	< 0.001
FINS, μU/mL	7.53 ± 3.67	6.56 ± 3.39	4.174	< 0.001
HOMA-IR	1.50 ± 0.80	1.27 ± 0.69	4.483	< 0.001
QUICKI	0.37 ± 0.03	0.38 ± 0.03	-4.483	< 0.001
Blood tests in the second trimester				
TC, mmol/L	6.35 ± 1.20	6.33 ± 1.06	0.270	0.788
TG, mmol/L	2.22 ± 0.79	2.12 ± 0.85	2.517	0.012
HDL-c, mmol/L	2.06 ± 0.42	2.08 ± 0.36	-0.869	0.385
LDL-c, mmol/L	3.64 ± 0.95	3.59 ± 0.74	0.566	0.571
HbAlc, %	5.05 ± 0.35	4.84 ± 0.30	8.152	< 0.001
FINS, μU/mL	9.42 ± 4.70	8.11 ± 3.77	4.488	< 0.001
HOMA-IR	1.95 ± 1.10	1.55 ± 0.77	5.851	< 0.001
QUICKI	0.35 ± 0.03	0.37 ± 0.03	-5.851	< 0.001
Oral glucose test result				
0h, mmol/L	4.56 ± 0.45	4.25 ± 0.30	9.740	< 0.001
1h, mmol/L	10.02 ± 1.39	7.43 ± 1.33	19.702	< 0.001
2h, mmol/L	8.80 ± 1.14	6.40 ± 1.04	20.930	< 0.001

BMI, body mass index; IVF, in vitro fertilization; TC, total cholesterol; TG, triglycerides; HDL-c, high-density lipoprotein cholesterol; LDL-c, low-density lipoprotein cholesterol; HbA1c, hemoglobin A1c; FPG, fasting plasma glucose; FINS, fasting insulin; HOMA-IR, homeostasis model assessment of insulin resistance; QUICKI, quantitative insulin sensitivity check index.

Compared to IR-H1 group, women had higher maternal BMI before pregnancy, first-trimester LDL-c, and both first- and second-trimester TG, HDL-c and FINS in IR-H2, IR-H3 and IR-H4 groups, respectively (all *P <*0.05) (**Table 2**). First-trimester TC was higher in IR-H2 group than that in IR-H1 group (**Table 2**). Comparison of lipid profiles and glucose metabolism indices between IR-Q1 group and IR-Q2, IR-Q3 and IR-Q4 groups demonstrated similar results (**Table 3**). We further compared lipid profiles among women with different IR statuses separately in first and second trimesters. The results showed that first-trimester TC and LDL-c, and first- and second-trimester TG and HDL-c were significantly different in those with high HOMA-IR compared to those without (**sTable 1**). Comparison of lipid profiles and glucose metabolism indices between women with and without low QUICKI also demonstrated the analogous results (**sTable 2**).

**Table 2:** Maternal characteristics, lipid profiles and glucose metabolism indices in different HOMA-IR subgroups.

Characteristics	IR-H1 N=778	IR-H2^a^ N=159	IR-H3^a^ N=148	IR-H4^a^ N=149
Maternal age, years	31.41 ± 4.24	32.34 ± 4.72*	31.29 ± 3.91	31.72 ± 4.44
BMI, kg/m^2^	20.42 ± 2.32	23.76 ± 3.30**	21.95 ± 3.02**	21.54 ± 2.30**
Conception by IVF, n (%)	133 (17.1)	25 (15.7)	30 (20.3)	33 (22.1)
Smoking, n (%)	5 (1.9)	3 (0.6)	3 (2.0)	1 (0.7)
Family history of diabetes mellitus, n (%)	33 (4.2)	12 (7.5)	10 (6.8)	11 (7.4)
Multiparous, n (%)	243 (31.2)	63 (39.6)*	52 (35.1)	54 (36.2)
Blood tests in the first trimester				
TC, mmol/L	4.91 ± 0.77	5.18 ± 0.83**	5.01 ± 0.82	5.03 ± 1.07
TG, mmol/L	1.26 ± 0.49	1.72 ± 0.67**	1.47 ± 0.53**	1.44 ± 0.45**
HDL-c, mmol/L	1.83 ± 0.32	1.71 ± 0.34**	1.73 ± 0.33**	1.72 ± 0.29**
LDL-c, mmol/L	2.71 ± 0.52	2.95 ± 0.58**	2.85 ± 0.56**	2.88 ± 0.77**
HbAlc, %	5.12 ± 0.29	5.21 ± 0.33**	5.12 ± 0.35	5.19 ± 0.31**
FPG, mmol/L	4.27 ± 0.32	4.49 ± 0.36**	4.57 ± 0.33**	4.31 ± 0.33
FINS, μU/mL	5.03 ± 1.63	12.21 ± 4.05**	10.16 ± 2.60**	6.48 ± 1.50**
Blood tests in the second trimester				
TC, mmol/L	6.33 ± 1.05	6.27 ± 1.11	6.34 ± 1.11	6.41 ± 1.25
TG, mmol/L	1.96 ± 0.72	2.54 ± 1.00**	2.34 ± 0.86**	2.42 ± 0.91**
HDL-c, mmol/L	2.11 ± 0.37	1.99 ± 0.35**	2.03 ± 0.36*	2.02 ± 0.35**
LDL-c, mmol/L	3.58 ± 0.73	3.56 ± 0.80	3.64 ± 0.76	3.70 ± 1.03
HbAlc, %	4.83 ± 0.30	5.04 ± 0.34**	4.86 ± 0.28	4.96 ± 0.34**
FINS, μU/mL	6.41 ± 1.87	14.78 ± 5.01**	7.76 ± 1.58**	12.24 ± 2.30**
Oral glucose test result				
0h, mmol/L	4.21 ± 0.30	4.60 ± 0.40**	4.33 ± 0.30**	4.52 ± 0.37**
1h, mmol/L	7.70 ± 1.58	8.80 ± 1.70**	7.80 ± 1.64	8.26 ± 1.84**
2h, mmol/L	6.66 ± 1.37	7.51 ± 1.43**	6.86 ± 1.33*	7.13 ± 1.43**

^a^ In comparison with IR-H1 group; *P < 0.05; **P < 0.01.

**Table 3:** Maternal characteristics, lipid profiles and glucose metabolism indices in different QUICKI subgroups.

Characteristics	IR-Q1 N=783	IR-Q2^a ^N=155	IR-Q3^a^ N=142	IR-Q4^a^ N=154
Maternal age, years	31.42 ± 4.24	32.30 ± 4.76*	31.25 ± 3.91	31.76 ± 4.41
BMI, kg/m^2^	20.43 ± 2.33	23.83 ± 3.31**	21.98 ± 3.04**	21.53 ± 2.27**
Conception by IVF, n (%)	134 (17.1)	25 (16.1)	29 (20.4)	33 (21.4)
Smoking, n (%)	5 (0.6)	3 (1.9)	3 (2.1)	1 (0.6)
Family history of diabetes mellitus, n (%)	34 (4.3)	11 (7.1)	9 (6.3)	12 (7.8)
Multiparous, n (%)	245 (31.3)	62 (40.0)*	50 (35.2)	55 (35.7)
Blood tests in the first trimester				
TC, mmol/L	4.91 ± 0.77	5.19 ± 0.84**	5.01 ± 0.83	5.02 ± 1.05
TG, mmol/L	1.26 ± 0.49	1.73 ± 0.68**	1.48 ± 0.54**	1.44 ± 0.45**
HDL-c, mmol/L	1.83 ± 0.32	1.71 ± 0.34**	1.73 ± 0.33**	1.72 ± 0.29**
LDL-c, mmol/L	2.71 ± 0.52	2.97 ± 0.58**	2.86 ± 0.56**	2.87 ± 0.76**
HbAlc, %	5.12 ± 0.29	5.20 ± 0.33**	5.11 ± 0.35	5.19 ± 0.31**
FPG, mmol/L	4.27 ± 0.32	4.49 ± 0.36**	4.58 ± 0.34**	4.31 ± 0.33
FINS, μU/mL	5.05 ± 1.65	12.32 ± 4.05**	10.24 ± 2.62**	6.51 ± 1.51**
Blood tests in the second trimester				
TC, mmol/L	6.32 ± 1.05	6.28 ± 1.12	6.35 ± 1.12	6.40 ± 1.24
TG, mmol/L	1.96 ± 0.72	2.55 ± 1.01**	2.35 ± 0.87**	2.41 ± 0.90**
HDL-c, mmol/L	2.11 ± 0.37	1.99 ± 0.35**	2.03 ± 0.37*	2.02 ± 0.35**
LDL-c, mmol/L	3.58 ± 0.73	3.56 ± 0.81	3.65 ± 0.76	3.69 ± 1.02
HbAlc, %	4.83 ± 0.30	5.05 ± 0.34**	4.85 ± 0.28	4.96 ± 0.33**
FINS, μU/mL	6.41 ± 1.86	14.88 ± 5.03**	7.78 ± 1.59**	12.19 ± 2.29**
Oral glucose test result				
FPG, mmol/L	4.21 ± 0.30	4.60 ± 0.40**	4.33 ± 0.30**	4.51 ± 0.37**
1-h PG, mmol/L	7.71 ± 1.58	8.82 ± 1.71**	7.77 ± 1.63	8.27 ± 1.82**
2-h PG, mmol/L	6.66 ± 1.37	7.53 ± 1.43**	6.84 ± 1.35	7.14 ± 1.43**

^a^ In comparison with IR-Q1 group; *P < 0.05; **P < 0.01.

### Correlation between lipid profiles, glucose metabolism indices and insulin indices

**Figure 1:**
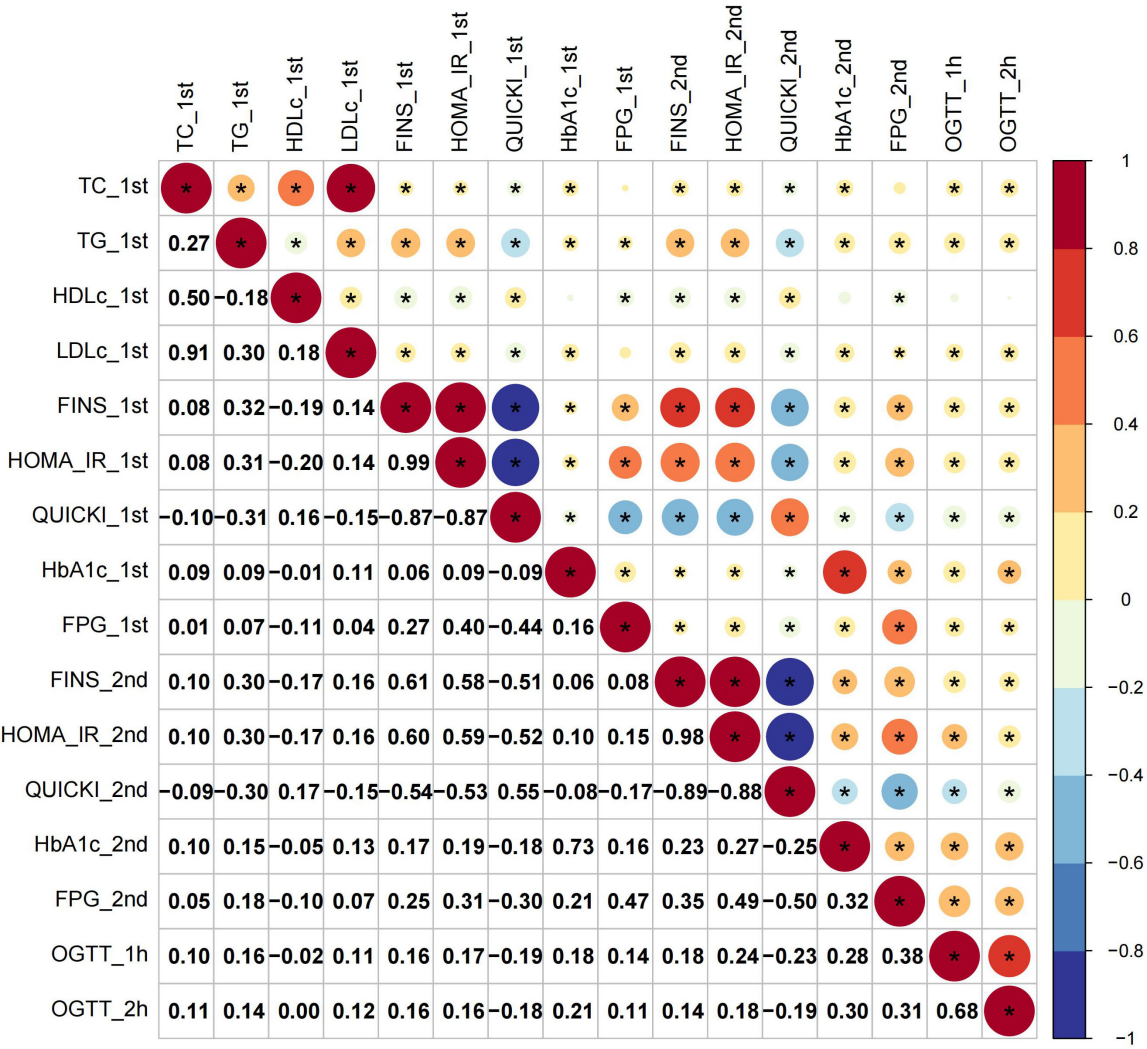
Correlations of first-trimester lipid profiles with glucose metabolism and insulin indices. Asterisk (*) in the circles denoted *P* < 0.05; _1st and _2nd denoted first and second trimester, respectively.

**Figure 2:**
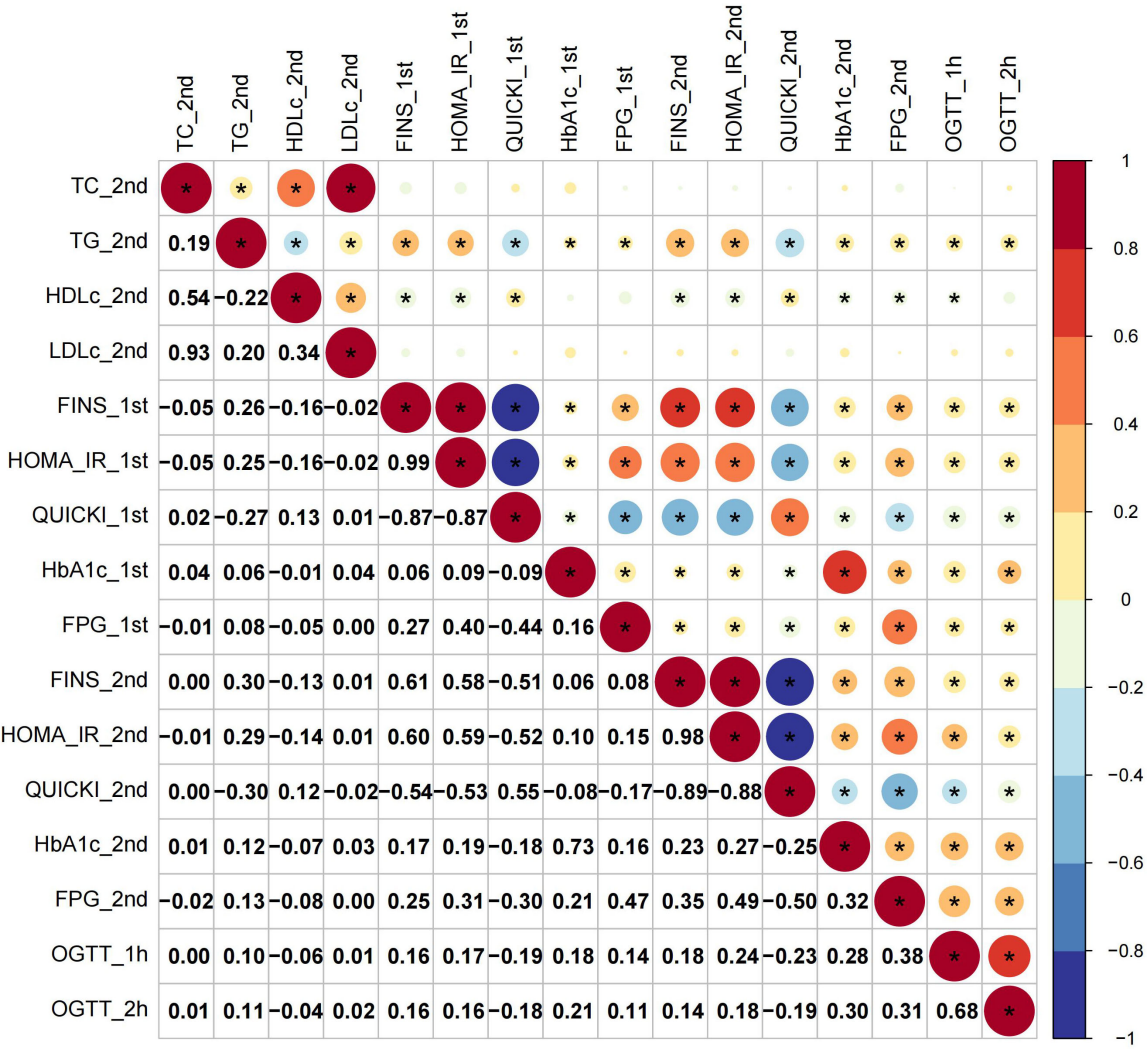
Correlations of second-trimester lipid profiles with glucose metabolism and insulin indices.

The first-trimester levels of TC, TG, LDL-c and HDL-c showed significant correlation to first- and second-trimester FINS, HOMA-IR and QUICKI (**Figure 1**). Among them TG had stronger connection to insulin indices (First trimester: FINS: r=0.32, HOMA-IR: r=0.31, QUICKI: r=-0.31; second trimester: FINS: r=0.30, HOMA-IR: r=0.30; QUICKI: r=-0.30; all *P* < 0.05) (**Figure 1**). The second-trimester levels of TG and HDL-c represented positive relationship to first- and second-trimester FINS, HOMA-IR and QUICKI (**Figure 2**). As shown in **Figure 2**, TG still had relatively higher connection to insulin indices (First trimester: FINS: r=0.26, HOMA-IR: r=0.25, QUICKI: r=-0.27; second trimester: FINS: r=0.30, HOMA-IR: r=0.29; QUICKI: r=-0.30; all *P* < 0.05).

For glucose metabolism indices, only rises in TG paralleled increases in all the glucose metabolism indices across the two trimesters (**Figures 1**, **2**). Overall, TG showed a more significant connection to insulin indices than to glucose metabolism indices. The relationship between first- and second-trimester lipid profiles were depicted in **sFigure 1**.

### Trends in lipid profiles and risk of GDM and IR

**Figure 3:**
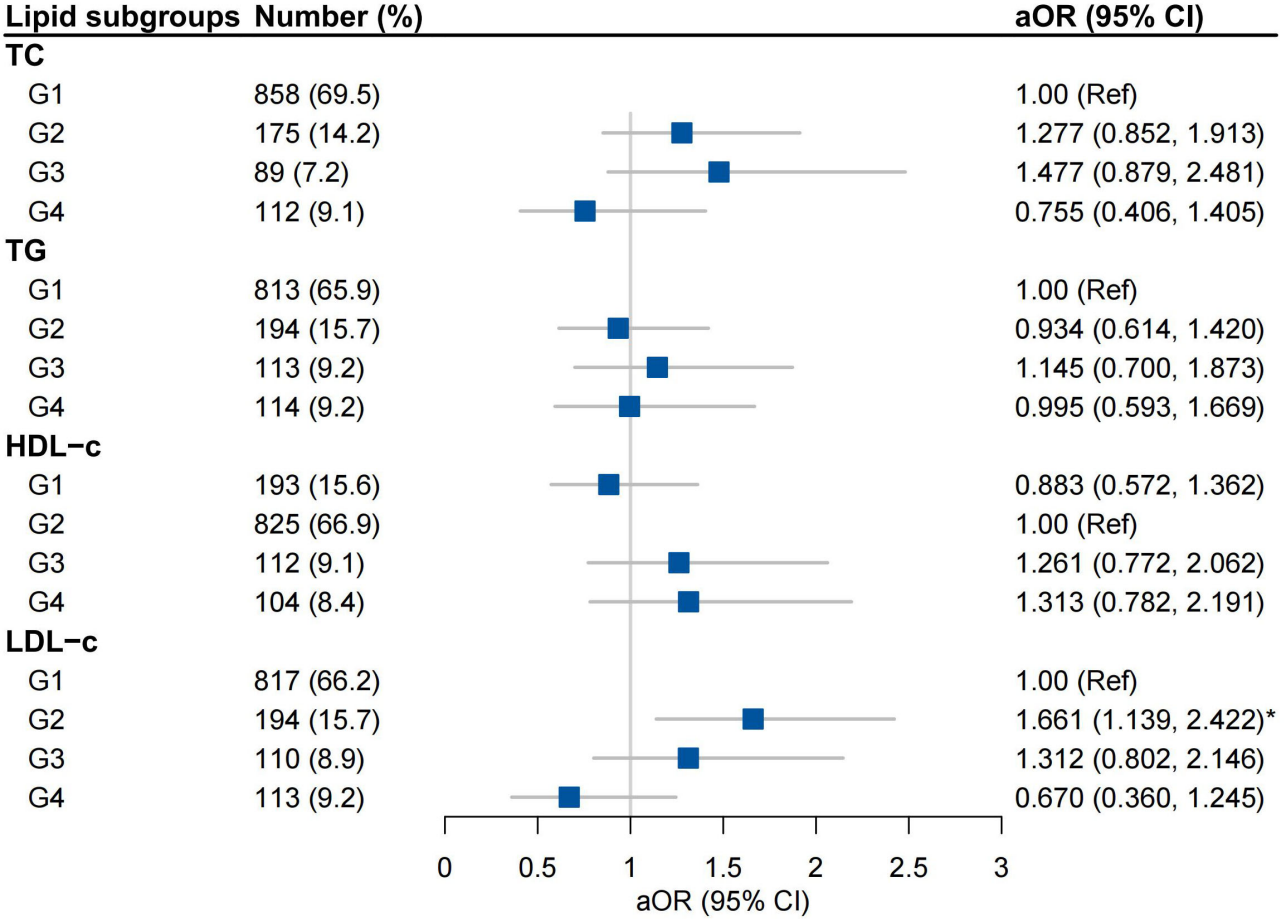
Associations between trends in lipid profiles and GDM Adjusted for Maternal age, BMI, Conception by IVF, Family history of diabetes mellitus, Smoking and Multiparous; G1, low-to-low group; G2, high-to-high group; G3, high-to-low group; G4 low-to-high group.

After adjustment for covariates, high-to-high (G2) LDL-c was an independent risk factor for GDM (aOR 1.661, 95% CI 1.139-2.422), but not high-to-low (G3) or low-to-high LDL-c (G4) group (**Figure 3**). Trends in TG were not associated with the incidence of GDM (**Figure 3**). Stratified analysis indicated that high-to-high LDL-c group contributed to risk of GDM in women with underweight and obese or overweight, not in women with normal weight (**sTable 3**).

**Figure 4:**
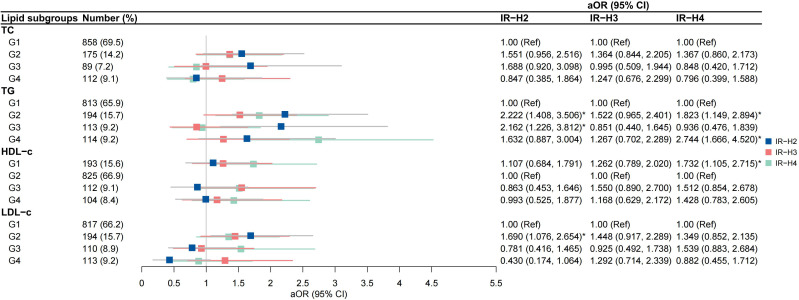
Associations between trends in lipid profiles and trends in insulin resistance calculated by HOMA-IR IR-H2, pregnancies with persistently high HOMA-IR in both the first and second trimesters; IR-H3 group, pregnancies with first-trimester high HOMA-IR alone; IR-H4 group, pregnancies with second-trimester high HOMA-IR alone.

**Figure 5:**
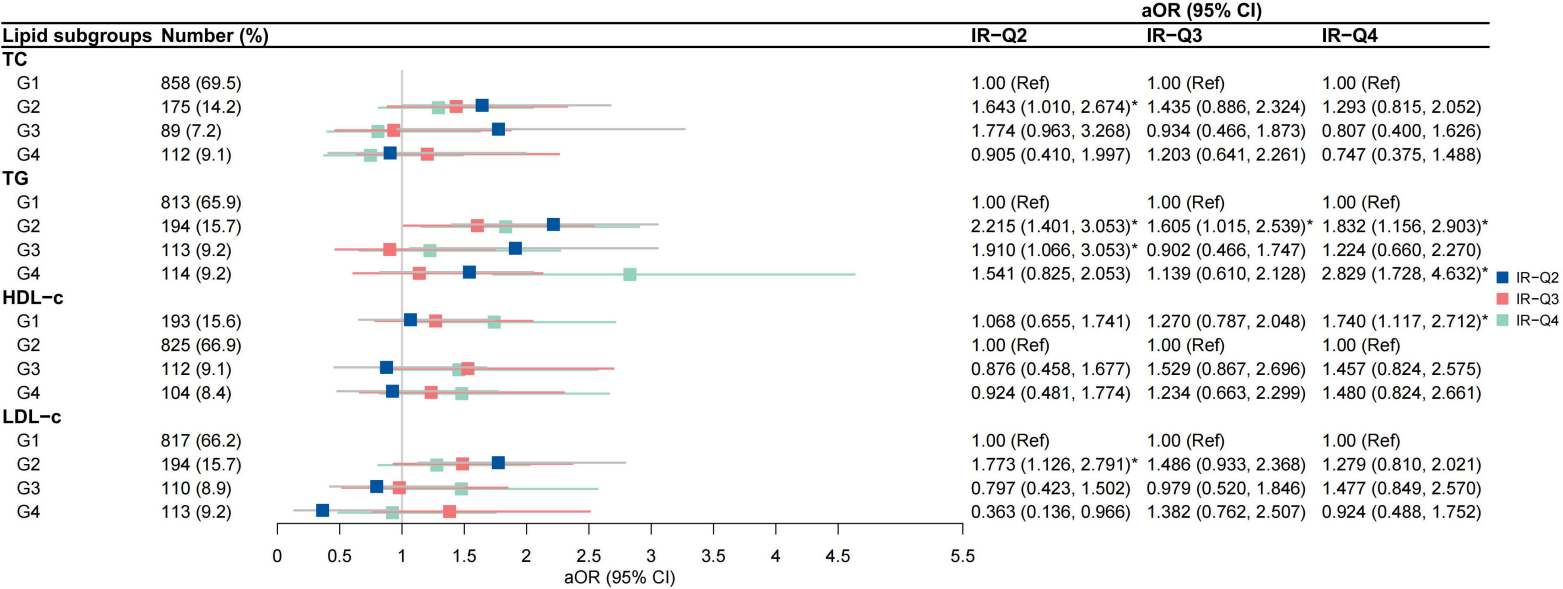
Associations between trends in lipid profiles and trends in insulin resistance calculated by QUICKI IR-Q2, pregnancies with persistently low QUICKI in both the first and second trimesters; IR-Q3 group, pregnancies with first-trimester low QUICKI alone; IR-Q4 group, pregnancies with second-trimester high low QUICKI alone.

High-to-high (G2) TG group was an independent risk factor for IR-H2 and IR-H4 (aOR 2.222, 95% CI 1.408-3.506; aOR 1.823, 95% CI 1.149-2.894, respectively) after the adjustment for covariates (**Figure 4**). High-to-low (G3) TG group was associated with IR-H2 (aOR 2.162, 95% CI 1.226-3.812), and low-to-high (G4) TG group was correlated with IR-H4 (aOR 2.744, 95% CI 1.666-4.520) (**Figure 4**). The associations of G2 group of TGs with IR-Q2 and IR-Q4 were also validated (**Figure 5**). Besides, G2 group of TGs increased the risk of IR-Q3 (aOR 1.605, 95% CI 1.015-2.539) (**Figure 5**). Low-to-low (G1) HDL-c demonstrated comparable risk of IR-H4 (aOR 1.732, 95% CI 1.105-2.715) and IR-Q4 (aOR 1.740, 95% CI 1.117-2.712). High-to-high (G2) LDL-c revealed comparable risk of IR-H1 (aOR 1.690, 95% CI 1.076-2.654) and IR-Q1 (aOR 1.773, 95% CI 1.126-2.791) (**Figures 4**, **5**). Stratified analysis showed significant association of high-to-high TG with persistent IR and second-trimester IR alone, respectively for women with normal weight, but not for women with underweight and overweight or obese (**sTables 4**, **5**).

## Discussion

This study has demonstrated that first, the concentration of first- and second-trimester lipid profiles are significantly different between women with and without GDM, and between women with and without IR; second, both first- and second-trimester TC, TG, and HDL-c are strongly correlated to first- and second-trimester insulin indices, while only TG has sustained correlation with glucose metabolism indices; third, persistently high TG is an independent risk factor for persistent IR and second-trimester IR alone.

The association between hypertriglyceridemia and GDM has been well elaborated. Recent meta-analysis has reported that among studies exploring the relationship between lipid profiles and GDM, TG is the most crucial with most included studies reporting higher TG levels in women with GDM (22). Further analysis has supported that higher TG levels in women with GDM occur in the first trimester and persist across pregnancy (22). Our study also has shown elevated TG levels in women with GDM in both first (1.47 ± 0.56 mmol/L vs 1.33 ± 0.53 mmol/L, *P* < 0.001) and second trimester (2.22 ± 0.79 mmol/L vs 2.12 ± 0.85 mmol/L, *P* < 0.001), compared to women without GDM. However, results are less consistent for the other lipids. Some evidence has shown elevated TC and LDL-c levels in women with GDM compared to those without. While a nested case-control study that has measured first- and second-trimester lipid profiles levels among 318 pregnant women conclude that there is no statistical difference in TC and LDL-c between women with GDM and those without (23). This conclusion is also supported by another meta-analysis (24). Our study has observed higher TC and LDL-c levels in women with GDM than those without in the first trimester but not in the second trimester. However, no significant difference in HDL-c has been found between women with and without GDM across two trimesters.

Consistent with previous research, TG levels strongly correlate with OGTT test results in both the first and second trimesters (16, 22). Nevertheless, trends in TG are not an independent risk for GDM in our study after adjusting for covariates. In stratified analysis, persistently high TG is associated with GDM in women with underweight (aOR 7.626, 95% CI 1.268-45.857), but the wide CI indicates that this result is not sufficiently powered. Several risk factors have been proposed to contribute to GDM, such as being overweight or obese before pregnancy, genetic factors, inflammatory factors and dyslipidemia (especially hypertriglyceridemia irrespective of the period in pregnancy) (8, 16, 25–27). A large-scale retrospective study in China has reported that persistently high TG levels (defined as above the 90th percentile of the population) in the first and third trimesters increase the risk of GDM (aOR 1.97, 95% CI 1.57-2.47), compared to those with persistently low TG levels throughout pregnancy (16). It seems that our results with respect to TG and risk of GDM are rather incompatible with existing conclusions. This inconsistency may be due to the one-step approach for screening GDM in our cohort, which is still under debate whether it will identify more women that are considered as low risk for GDM compared to the two-step approach (28). The lower definition thresholds may lead to overdiagnosis and thus reduce the efficacy of TG to detect the risk for GDM.

Hypertriglyceridemia is an outstanding reflection of insulin resistance. It is an essential criterion for diagnosing metabolic syndrome (29) and both of which are known to be associated with adverse pregnancy outcomes, such as hypertensive disorders of pregnancy (12, 16, 30), preterm labor (13) and LGA (12, 13, 16, 31); and with long-term risk for cardiovascular disease (32, 33). The relationship between elevated TG levels and increased risk of IR has been clearly stated in non-pregnant individuals (29, 34, 35). Nevertheless, only a few studies have reported such a correlation in pregnant population in the first (9) or second trimester (36, 37). The current study has directly indicated the sustained correlation between TG and insulin indices in the first and second trimesters. Also, it has been denoted that high-to-high TG is an independent risk factor for persistent IR and second-trimester IR alone. In stratified analysis, the associations of persistently high TG with persistent IR and second-trimester IR alone exist in women with normal weight (IR-H2: aOR 2.470, 95% CI 1.391-4.383; IR-H4: aOR 2.389, 95% CI 1.437-3.969; IR-Q2: aOR 2.487, 95% CI 1.398-4.422; IR-Q4: aOR 2.382, 95% CI 1.436-3.950), but not in women with underweight and overweight or obesity. Another retrospective study including 2647 GDM women in China has demonstrated the connections between HOMA-IR and adverse pregnancy outcomes (13). However, stratified analysis of their study also fails to support such results in women with underweight, overweight, or obesity. The authors have claimed that they could not differentiate whether nonsignificant association is contributed by early lifestyle interventions in obese women. In our cohort, no interventions have been applied to overweight or obese women attending for first antenatal visit, but we have not recorded their lifestyle such as physical activities and dietary patterns before and during pregnancy. Therefore, further research is needed to investigate whether the underlying pathogenesis of IR during pregnancy is discordant between women with overweight or obese and with normal weight.

Both high-to-high TG group and high-to-low TG group are associated with the risk of persistent IR, implying that no matter the TG levels in the second trimester, pregnant women would suffer an increased risk of persistent IR so long as the TG levels are high in their first trimester. This result emphasizes the importance of lowering lipid levels in early pregnancy or even before conception to prevent the development of persistent IR in women with first-trimester IR. On the other hand, high-to-high TG group and low-to-high TG group are associated with the risk of women who have no IR in the first trimester but develop IR afterward, whereas high-to-low TG group is not. Consequently, for women without first-trimester IR, it is still reasonable to lower their TG levels in the first and second trimesters to reduce the risk of the later development of IR. Since plasma insulin concentration is not a routine blood test during antenatal visits in most care centers, TG may be additionally used as a surrogate estimate of insulin resistance during pregnancy. The associations between longitudinal trends in TG and IR in our study highlight the benefit of lowering lipid levels in early and middle pregnancy to prevent IR.

The main strength of our study included the synchronous screening of first- and second-trimester lipid profiles, glucose metabolism and insulin indices in the same population. Outcome variables were analyzed in continuous and categorical forms to strengthen the robustness of the results. The main baseline characteristics that may contribute to the outcomes of our cohort were almost completely extracted. There are also some limitations of our study. We only collected first- and second-trimester blood tests, making it difficult to depict the trends in lipid profiles and insulin indices throughout the pregnancy. In line with previous studies, we have confirmed the relationships between TG and IR in the first and second trimesters, therefore more effort is needed to identify the association between lipid profiles and insulin indices in the third trimester. The setting of single center and exclusion of multiple pregnancies limit the generalizability of our results. The third limitation was that the comparison of maternal and fetal outcomes between lipid subgroups and IR subgroups was lacking, albeit the effect of dyslipidemia and IR on adverse outcomes has been widely discussed in previous research. Finally, due to the study design, we did not collect other factors which may influence maternal lipid levels, including thyroid hormone, weight gain during pregnancy and factors related to hypercoagulability such as antenatal hospital admission and family history of venous thromboembolism.

In conclusion, our results suggest that TG has a sustained correlation with insulin indices and glucose metabolism indices in both the first and second trimesters. In addition, persistently high TG is an independent risk factor for persistent IR and second-trimester IR alone. For women with first-trimester IR, it is still important to lower their lipid levels in early pregnancy or even before conception to prevent the development of persistent IR. For women without first-trimester IR, it is still reasonable to lower TG levels in the first and second trimesters to reduce the risk of the later development of IR. These results together highlight the benefit of lowering TG levels in early and middle pregnancy to prevent the development of IR.

## Data availability statement

The original contributions presented in the study are included in the article/**Supplementary Material**. Further inquiries can be directed to the corresponding authors.

## Ethics statement

Approval for the study was obtained from the Independent Ethics Committee for Clinical Research and Animal Trials of First Affiliated Hospital of Sun Yat-sen University in Guangzhou. All eligible women were given written information about the study and those who agreed to participate provided written informed consent.

## Author contributions

LS, DW and YH performed the statistical analysis and wrote the manuscript. LY and CZ contributed to the planning of the study, collected study data and reviewed the manuscript. SZ and SC collected the study data. ZW and HC designed the study and revised the manuscript critically. All authors contributed to the article and approved the submitted version.

## References

[1] Juan J Yang H. Prevalence, prevention, and lifestyle intervention of gestational diabetes mellitus in China. Int J Environ Res Public Health (2020) 17(24):9517. doi: 10.3390/ijerph17249517 33353136 PMC7766930

[2] Nguyen CL Pham NM Binns CW Duong DV Lee AH. Prevalence of gestational diabetes mellitus in Eastern and southeastern Asia: A systematic review and meta-analysis. J Diabetes Res (2018) 2018:6536974. doi: 10.1155/2018/6536974 29675432 PMC5838488

[3] Zhu H Zhao Z Xu J Chen Y Zhu Q Zhou L. The prevalence of gestational diabetes mellitus before and after the implementation of the universal two-child policy in China. Front Endocrinol (2022) 13:960877. doi: 10.3389/fendo.2022.960877 PMC9433653 36060951

[4] Ye W Luo C Huang J Li C Liu Z Liu F. Gestational diabetes mellitus and adverse pregnancy outcomes: systematic review and meta-analysis. BMJ (2022) 377:e067946. doi: 10.1136/bmj-2021-067946 35613728 PMC9131781

[5] Metzger BE Lowe LP Dyer AR Trimble ER Chaovarindr U Coustan DR. Hyperglycemia and adverse pregnancy outcomes. N Engl J Med (2008) 358(19):1991–2002. doi: 10.1056/NEJMoa0707943 18463375

[6] Lowe WL Jr. Scholtens DM Kuang A Linder B Lawrence JM Lebenthal Y. Hyperglycemia and adverse pregnancy outcome follow-up study (HAPO FUS): Maternal gestational diabetes mellitus and childhood glucose metabolism. Diabetes Care (2019) 42(3):372–80. doi: 10.2337/dc18-1646 PMC6385693 30655380

[7] Li Z Cheng Y Wang D Chen H Chen H Ming WK. Incidence rate of type 2 diabetes mellitus after gestational diabetes mellitus: A systematic review and meta-analysis of 170,139 women. J Diabetes Res (2020) 2020:3076463. doi: 10.1155/2020/3076463 32405502 PMC7204113

[8] McIntyre HD Catalano P Zhang C Desoye G Mathiesen ER Damm P. Gestational diabetes mellitus. Nat Rev Dis Primers (2019) 5(1):47. doi: 10.1038/s41572-019-0098-8 31296866

[9] Song S Zhang Y Qiao X Duo Y Xu J Peng Z. HOMA-IR as a risk factor of gestational diabetes mellitus and a novel simple surrogate index in early pregnancy. Int J Gynaecol Obstet (2022) 157(3):694–701. doi: 10.1002/ijgo.13905 34449903

[10] Alptekin H Çizmecioğlu A Işık H Cengiz T Yildiz M Iyisoy MS. Predicting gestational diabetes mellitus during the first trimester using anthropometric measurements and HOMA-IR. J Endocrinol Invest (2016) 39(5):577–83. doi: 10.1007/s40618-015-0427-z 26754418

[11] Grewal E Kansara S Kachhawa G Ammini AC Kriplani A Aggarwal N. Prediction of gestational diabetes mellitus at 24 to 28 weeks of gestation by using first-trimester insulin sensitivity indices in Asian Indian subjects. Metabolism (2012) 61(5):715–20. doi: 10.1016/j.metabol.2011.10.009 22146095

[12] Lin J Jin H Chen L. Associations between insulin resistance and adverse pregnancy outcomes in women with gestational diabetes mellitus: a retrospective study. BMC Pregnancy Childbirth (2021) 21(1):526. doi: 10.1186/s12884-021-04006-x 34301212 PMC8306365

[13] Sun YY Juan J Xu QQ Su RN Hirst JE Yang HX. Increasing insulin resistance predicts adverse pregnancy outcomes in women with gestational diabetes mellitus. J Diabetes (2020) 12(6):438–46. doi: 10.1111/1753-0407.13013 31808991

[14] Samuel VT Shulman GI. Mechanisms for insulin resistance: common threads and missing links. Cell (2012) 148(5):852–71. doi: 10.1016/j.cell.2012.02.017 PMC3294420 22385956

[15] Kampmann U Knorr S Fuglsang J Ovesen P. Determinants of maternal insulin resistance during pregnancy: An updated overview. J Diabetes Res (2019) 2019:5320156. doi: 10.1155/2019/5320156 31828161 PMC6885766

[16] Xue RH Wu DD Zhou CL Chen L Li J Li ZZ. Association of high maternal triglyceride levels early and late in pregnancy with adverse outcomes: A retrospective cohort study. J Clin Lipidol (2021) 15(1):162–72. doi: 10.1016/j.jacl.2020.10.001 33144084

[17] Xi F Chen H Chen Q Chen D Chen Y Sagnelli M. Second-trimester and third-trimester maternal lipid profiles significantly correlated to LGA and macrosomia. Arch Gynecol Obstet (2021) 304(4):885–94. doi: 10.1007/s00404-021-06010-0 33651156

[18] Metzger BE Gabbe SG Persson B Buchanan TA Catalano PA Damm P. International association of diabetes and pregnancy study groups recommendations on the diagnosis and classification of hyperglycemia in pregnancy. Diabetes Care (2010) 33(3):676–82. doi: 10.2337/dc09-1848 PMC2827530 20190296

[19] American Diabetes Association. 2. classification and diagnosis of diabetes: Standards of medical care in diabetes–2021. Diabetes Care (2020) 44(Supplement_1):S15–33. doi: 10.2337/dc21-S002 33298413

[20] Chinese Medical Association. Diagnosis and therapy guideline of pregnancy with diabetes mellitus. Chin J Obstet Gynecol (2014) 49(8):561–9. doi: 10.3760/cma.j.issn.0529-567X.2014.08.001 25354853

[21] Alberti KG Zimmet PZ. Definition, diagnosis and classification of diabetes mellitus and its complications. part 1: diagnosis and classification of diabetes mellitus provisional report of a WHO consultation. Diabetes Med (1998) 15(7):539–53. doi: 10.1002/(sici)1096-9136(199807)15:7<539::Aid-dia668>3.0.Co;2-s 9686693

[22] Hu J Gillies CL Lin S Stewart ZA Melford SE Abrams KR. Association of maternal lipid profile and gestational diabetes mellitus: A systematic review and meta-analysis of 292 studies and 97,880 women. EClinicalMedicine (2021) 34:100830. doi: 10.1016/j.eclinm.2021.100830 33997732 PMC8102708

[23] Bao W Dar S Zhu Y Wu J Rawal S Li S. Plasma concentrations of lipids during pregnancy and the risk of gestational diabetes mellitus: A longitudinal study. J Diabetes (2018) 10(6):487–95. doi: 10.1111/1753-0407.12563 PMC5837900 28436169

[24] Ryckman K Spracklen C Smith C Robinson J Saftlas A. Maternal lipid levels during pregnancy and gestational diabetes: a systematic review and meta-analysis. BJOG (2015) 122(5):643–51. doi: 10.1111/1471-0528.13261 25612005

[25] Wu YT Zhang CJ Mol BW Kawai A Li C Chen L. Early prediction of gestational diabetes mellitus in the Chinese population *via* advanced machine learning. J Clin Endocrinol Metab (2021) 106(3):e1191–e205. doi: 10.1210/clinem/dgaa899 PMC7947802 33351102

[26] Zhu H He D Liang N Lai A Zeng J Yu H. High serum triglyceride levels in the early first trimester of pregnancy are associated with gestational diabetes mellitus: A prospective cohort study. J Diabetes Investig (2020) 11(6):1635–42. doi: 10.1111/jdi.13273 PMC7610113 32281298

[27] Zheng T Ye W Wang X Li X Zhang J Little J. A simple model to predict risk of gestational diabetes mellitus from 8 to 20 weeks of gestation in Chinese women. BMC Pregnancy Childbirth (2019) 19(1):252. doi: 10.1186/s12884-019-2374-8 31324151 PMC6642502

[28] Hillier TA Pedula KL Ogasawara KK Vesco KK Oshiro CES Lubarsky SL. A pragmatic, randomized clinical trial of gestational diabetes screening. N Engl J Med (2021) 384(10):895–904. doi: 10.1056/NEJMoa2026028 33704936 PMC9041326

[29] Eckel RH Grundy SM Zimmet PZ. The metabolic syndrome. Lancet (2005) 365(9468):1415–28. doi: 10.1016/s0140-6736(05)66378-7 15836891

[30] Jin WY Lin SL Hou RL Chen XY Han T Jin Y. Associations between maternal lipid profile and pregnancy complications and perinatal outcomes: a population-based study from China. BMC Pregnancy Childbirth (2016) 16:60. doi: 10.1186/s12884-016-0852-9 27000102 PMC4802610

[31] Bever AM Mumford SL Schisterman EF Sjaarda L Perkins NJ Gerlanc N. Maternal preconception lipid profile and gestational lipid changes in relation to birthweight outcomes. Sci Rep (2020) 10(1):1374. doi: 10.1038/s41598-019-57373-z 31992758 PMC6987205

[32] McLaughlin T Allison G Abbasi F Lamendola C Reaven G. Prevalence of insulin resistance and associated cardiovascular disease risk factors among normal weight, overweight, and obese individuals. Metabolism (2004) 53(4):495–9. doi: 10.1016/j.metabol.2003.10.032 15045698

[33] Zawiejska A Wróblewska-Seniuk K Gutaj P Kippen J Gomulska A Wender-Ozegowska E. Markers of maternal insulin resistance and lipid ratios measured in early pregnancy are related to adverse fetomaternal outcomes in women treated for hyperglycemia detected in early pregnancy-data from a retrospective cohort study. J Clin Med (2022) 11(7):1777. doi: 10.3390/jcm11071777 35407384 PMC8999957

[34] Lin D Qi Y Huang C Wu M Wang C Li F. Associations of lipid parameters with insulin resistance and diabetes: A population-based study. Clin Nutr (2018) 37(4):1423–9. doi: 10.1016/j.clnu.2017.06.018 28673690

[35] McLaughlin T Abbasi F Cheal K Chu J Lamendola C Reaven G. Use of metabolic markers to identify overweight individuals who are insulin resistant. Ann Intern Med (2003) 139(10):802–9. doi: 10.7326/0003-4819-139-10-200311180-00007 14623617

[36] Poveda NE Garcés MF Darghan AE Jaimes SAB Sánchez EP Díaz-Cruz LA. Triglycerides/Glucose and Triglyceride/High-density lipoprotein cholesterol indices in normal and preeclamptic pregnancies: A longitudinal study. Int J Endocrinol (2018) 2018:8956404. doi: 10.1155/2018/8956404 30158976 PMC6109518

[37] Dinh Le T Minh Bui T Hien Vu T Phi Thi Nguyen N Thanh Thi Tran H Nguyen ST. Insulin resistance in gestational diabetes mellitus and its association with anthropometric fetal indices. Clin Med Insights Endocrinol Diabetes (2022) 15:11795514221098403. doi: 10.1177/11795514221098403 35601878 PMC9121510

