# Supplementary figures and images for: Longitudinal trends in lipid profiles during pregnancy: Association with gestational diabetes mellitus and longitudinal trends in insulin indices
Source: Front Endocrinol (Lausanne). 2023 Jan 13;13:1080633. doi: 10.3389/fendo.2022.1080633 (PMC9880552; doi:10.3389/fendo.2022.1080633)

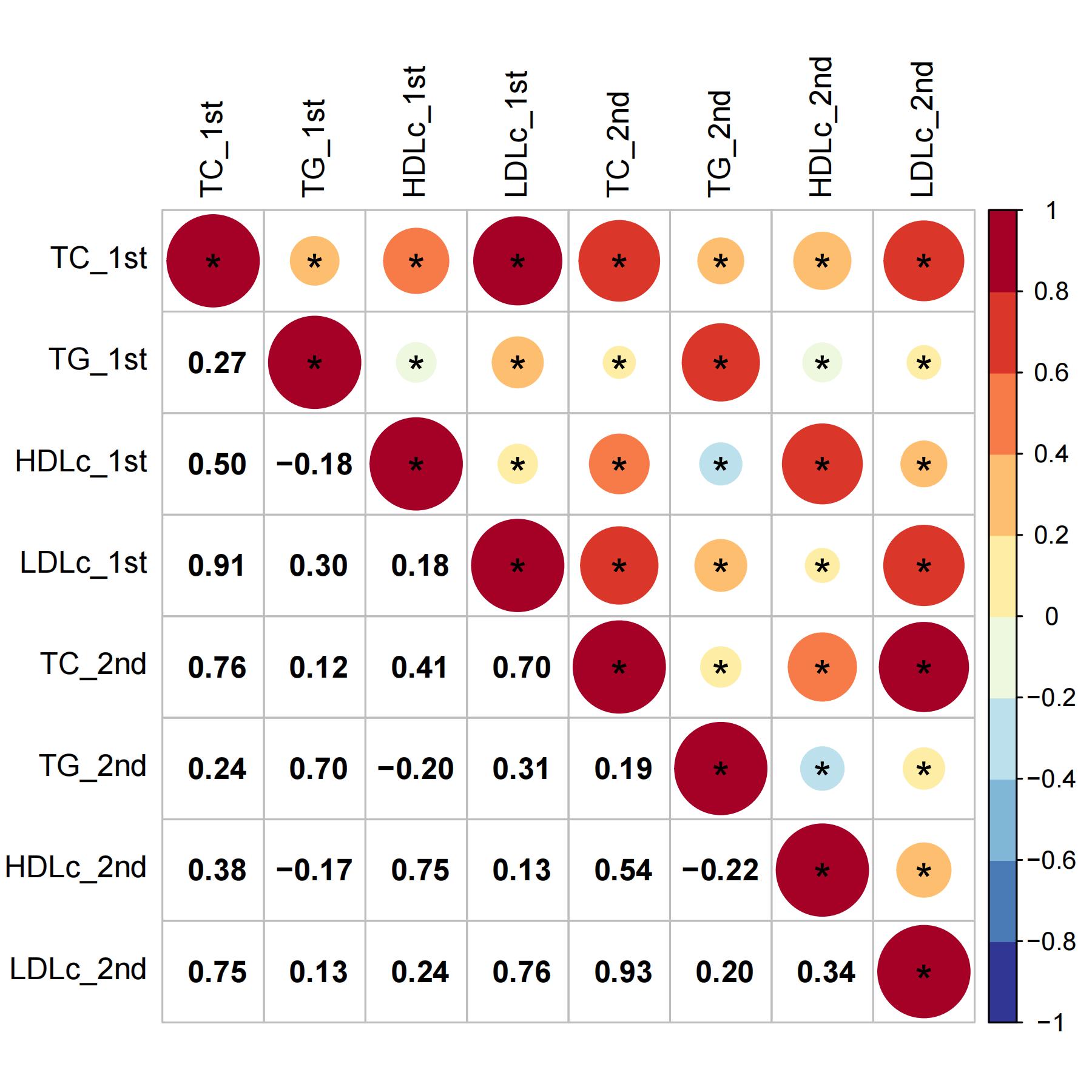

Supplement: Supplementary file 1 [file Image_1.jpeg]
