# Supplementary material for: Longitudinal trends in lipid profiles during pregnancy: Association with gestational diabetes mellitus and longitudinal trends in insulin indices
Source: Front Endocrinol (Lausanne). 2023 Jan 13;13:1080633. doi: 10.3389/fendo.2022.1080633 (PMC9880552; doi:10.3389/fendo.2022.1080633)
Supplement: Supplementary file 2 [file DataSheet_1.docx]

Supplementary Material

# Supplementary Tables

**sTable 1 Maternal characteristics, lipid profiles and glucose metabolism indices in women with high and low HOMA-IR**

| **Characteristics** | **First trimester** | | | **Second trimester** | | |
| --- | --- | --- | --- | --- | --- | --- |
|  | **High HOMA-IR**  N=307 | **Low HOMA-IR**  N=927 | ***P* value** | **High HOMA-IR**  N=308 | **Low HOMA-IR**  N=926 | ***P* value** |
| Maternal age, years | 31.83 ± 4.37 | 31.46 ± 4.27 | 0.196 | 32.04 ± 4.59 | 31.39 ± 4.19 | 0.041 |
| BMI, kg/m^2^ | 22.89 ± 3.29 | 20.60 ± 2.36 | < 0.001 | 22.69 ± 3.06 | 20.66 ± 2.51 | < 0.001 |
| Conception by IVF, n (%) | 55 (17.9) | 166 (17.9) | 0.997 | 58 (18.8) | 163 (17.6) | 0.626 |
| Smoking, n (%) | 6 (2.0) | 6 (0.6) | 0.092 | 4 (1.3) | 8 (0.9) | 0.735 |
| Family history of diabetes mellitus, n (%) | 22 (7.2) | 44 (4.7) | 0.102 | 23 (7.5) | 43 (4.6) | 0.056 |
| Multiparous, n (%) | 115 (37.5) | 297 (32.0) | 0.081 | 117 (38.0) | 295 (31.9) | 0.048 |
| **Blood tests at first trimester** | | | | | | |
| TC, mmol/L | 5.10 ± 0.83 | 4.93 ± 0.82 | 0.001 | 5.11 ± 0.96 | 4.93 ± 0.78 | 0.004 |
| TG, mmol/L | 1.60 ± 0.62 | 1.29 ± 0.49 | < 0.001 | 1.58 ± 0.59 | 1.29 ± 0.50 | < 0.001 |
| HDL-c, mmol/L | 1.72 ± 0.33 | 1.81 ± 0.32 | < 0.001 | 1.72 ± 0.32 | 1.81 ± 0.32 | < 0.001 |
| LDL-c, mmol/L | 2.91 ± 0.57 | 2.73 ± 0.57 | < 0.001 | 2.92 ± 0.67 | 2.73 ± 0.53 | < 0.001 |
| HbAlc, % | 5.16 ± 0.34 | 5.13 ± 0.29 | 0.179 | 5.20 ± 0.32 | 5.12 ± 0.30 | < 0.001 |
| FPG, mmol/L | 4.53 ± 0.35 | 4.27 ± 0.32 | < 0.001 | 4.40 ± 0.36 | 4.32 ± 0.34 | < 0.001 |
| FINS, μU/mL | 11.22 ± 3.58 | 5.26 ± 1.70 | < 0.001 | 9.44 ± 4.21 | 5.85 ± 2.62 | < 0.001 |
| **Blood tests at second trimester** | | | | | | |
| TC, mmol/L | 6.30 ± 1.11 | 6.34 ± 1.08 | 0.610 | 6.34 ± 1.18 | 6.33 ± 1.06 | 0.938 |
| TG, mmol/L | 2.44 ± 0.94 | 2.04 ± 0.78 | < 0.001 | 2.48 ± 0.96 | 2.02 ± 0.76 | < 0.001 |
| HDL-c, mmol/L | 2.01 ± 0.36 | 2.09 ± 0.37 | 0.001 | 2.00 ± 0.35 | 2.10 ± 0.37 | < 0.001 |
| LDL-c, mmol/L | 3.60 ± 0.78 | 3.60 ± 0.78 | 0.919 | 3.62 ± 0.92 | 3.59 ± 0.73 | 0.939 |
| HbAlc, % | 4.95 ± 0.33 | 4.85 ± 0.31 | < 0.001 | 5.00 ± 0.34 | 4.84 ± 0.30 | < 0.001 |
| FINS, μU/mL | 11.40 ± 5.15 | 7.35 ± 2.90 | < 0.001 | 13.55 ± 4.13 | 6.63 ± 1.89 | < 0.001 |
| **Oral glucose test result** | | | | | | |
| FPG, mmol/L | 4.47 ± 0.38 | 4.26 ± 0.33 | < 0.001 | 4.56 ± 0.39 | 4.23 ± 0.30 | < 0.001 |
| 1-h PG, mmol/L | 8.32 ± 1.74 | 7.80 ± 1.64 | < 0.001 | 8.54 ± 1.79 | 7.72 ± 1.59 | < 0.001 |
| 2-h PG, mmol/L | 7.20 ± 1.42 | 6.74 ± 1.39 | < 0.001 | 7.33 ± 1.44 | 6.70 ± 1.37 | < 0.001 |

BMI, body mass index; IVF, in vitro fertilization; TC, total cholesterol; TG, triglycerides; HDL-c, high-density lipoprotein cholesterol; LDL-c, low-density lipoprotein cholesterol; HbA1c, hemoglobin A1c; FPG, fasting plasma glucose; FINS, fasting insulin; HOMA-IR, homeostasis model assessment of insulin resistance; QUICKI, quantitative insulin sensitivity check index.

**sTable 2 Maternal characteristics, lipid profiles and glucose metabolism indices in women with low and high QUICKI**

| **Characteristics** | **First trimester** | | | **Second trimester** | | |
| --- | --- | --- | --- | --- | --- | --- |
|  | **Low QUICKI**  N=297 | **High QUICKI**  N=937 | ***P* value** | **Low QUICKI**  N=309 | **High QUICKI**  N=925 | ***P* value** |
| Maternal age, years | 31.80 ± 4.40 | 31.47 ± 4.26 | 0.278 | 32.03 ± 4.59 | 31.39 ± 4.19 | 0.048 |
| BMI, kg/m^2^ | 22.94 ± 3.31 | 20.61 ± 2.35 | < 0.001 | 22.68 ± 3.06 | 20.66 ± 2.51 | 0.000 |
| Conception by IVF, n (%) | 54 (18.2) | 167 (17.8) | 0.888 | 309 (18.8) | 925 (17.6) | 0.648 |
| Smoking, n (%) | 6 (2.0) | 6 (0.6) | 0.076 | 4 (1.3) | 8 (0.9) | 0.740 |
| Family history of diabetes mellitus, n (%) | 20 (6.7) | 46 (4.9) | 0.223 | 23 (7.4) | 43 (4.6) | 0.059 |
| Multiparous, n (%) | 112 (37.7) | 300 (32.0) | 0.070 | 117 (37.9) | 295 (31.9) | 0.054 |
| **Blood tests at first trimester** | | | | | | |
| TC, mmol/L | 5.10 ± 0.84 | 4.93 ± 0.82 | 0.001 | 5.11 ± 0.95 | 4.93 ± 0.78 | 0.004 |
| TG, mmol/L | 1.61 ± 0.63 | 1.29 ± 0.50 | < 0.001 | 1.59 ± 0.59 | 1.29 ± 0.50 | < 0.001 |
| HDL-c, mmol/L | 1.72 ± 0.34 | 1.81 ± 0.32 | < 0.001 | 1.72 ± 0.32 | 1.81 ± 0.32 | < 0.001 |
| LDL-c, mmol/L | 2.91 ± 0.57 | 2.73 ± 0.57 | < 0.001 | 2.92 ± 0.67 | 2.73 ± 0.53 | < 0.001 |
| HbAlc, % | 5.16 ± 0.34 | 5.13 ± 0.29 | 0.264 | 5.20 ± 0.32 | 5.12 ± 0.30 | < 0.001 |
| FPG, mmol/L | 4.53 ± 0.35 | 4.28 ± 0.32 | < 0.001 | 4.40 ± 0.36 | 4.32 ± 0.34 | < 0.001 |
| FINS, μU/mL | 11.33 ± 3.59 | 5.29 ± 1.71 | < 0.001 | 9.42 ± 4.22 | 5.85 ± 2.62 | < 0.001 |
| **Blood tests at second trimester** | | | | | | |
| TC, mmol/L | 6.31 ± 1.12 | 6.34 ± 1.08 | 0.741 | 6.34 ± 1.18 | 6.33 ± 1.06 | 0.912 |
| TG, mmol/L | 2.46 ± 0.95 | 2.04 ± 0.77 | < 0.001 | 2.48 ± 0.96 | 2.02 ± 0.76 | < 0.001 |
| HDL-c, mmol/L | 2.00 ± 0.36 | 2.09 ± 0.37 | < 0.001 | 2.00 ± 0.35 | 2.10 ± 0.37 | < 0.001 |
| LDL-c, mmol/L | 3.60 ± 0.78 | 3.59 ± 0.78 | 0.786 | 3.62 ± 0.92 | 3.59 ± 0.73 | 0.957 |
| HbAlc, % | 4.95 ± 0.33 | 4.86 ± 0.31 | < 0.001 | 5.00 ± 0.34 | 4.84 ± 0.30 | < 0.001 |
| FINS, μU/mL | 11.49 ± 5.20 | 7.36 ± 2.89 | < 0.001 | 13.54 ± 4.13 | 6.62 ± 1.89 | < 0.001 |
| **Oral glucose test result** | | | | | | |
| FPG, mmol/L | 4.47 ± 0.38 | 4.26 ± 0.33 | < 0.001 | 4.56 ± 0.39 | 4.23 ± 0.30 | < 0.001 |
| 1-h PG, mmol/L | 8.32 ± 1.75 | 7.80 ± 1.64 | < 0.001 | 8.54 ± 1.79 | 7.72 ± 1.59 | < 0.001 |
| 2-h PG, mmol/L | 7.20 ± 1.43 | 6.74 ±1.39 | < 0.001 | 7.33 ± 1.44 | 6.69 ± 1.37 | < 0.001 |

**sTable 3 Association of trends in lipid profiles with GDM in different BMI subgroups**

| **Lipid subgroups** | **Underweight** | **Normal weight** | **Overweight or obesity** |
| --- | --- | --- | --- |
|  | **aOR (95% CI)^#^** | **aOR (95% CI) ^#^** | **aOR (95% CI) ^#^** |
| TC |  |  |  |
| G1 | 1.00 (Ref) | 1.00 (Ref) | 1.00 (Ref) |
| G2 | 11.444 (2.966, 44.154) | 1.013 (0.625, 1.644) | 1.202 (0.407, 3.550) |
| G3 | 3.640 (0.354, 37.408) | 0.981 (0.497, 1.939) | 4.433 (1.506, 13.055)* |
| G4 | 3.289 (0.720, 15.028) | 0.619 (0.295, 1.297) | 0.468 (0.049, 4.440) |
| TG |  |  |  |
| G1 | 1.00 (Ref) | 1.00 (Ref) | 1.00 (Ref) |
| G2 | 7.626 (1.268, 45.857)* | 0.791 (0.471, 1.328) | 1.147 (0.490, 2.684) |
| G3 | 0.692 (0.073, 6.571) | 1.023 (0.549, 1.905) | 1.933 (0.718, 5.200) |
| G4 | 3.191 (0.568, 17.921) | 0.817 (0.438, 1.524) | 2.307 (0.662, 8.034) |
| HDL-c |  |  |  |
| G1 | 0.446 (0.051, 3.922) | 0.960 (0.569, 1.620) | 0.844 (0.348, 2.049) |
| G2 | 1.00 (Ref) | 1.00 (Ref) | 1.00 (Ref) |
| G3 | 0.776 (0.119, 5.080) | 1.183 (0.647, 2.163) | 1.748 (0.563, 5.428) |
| G4 | NA | 1.927 (1.052, 3.531) | 0.870 (0.299, 2.529) |
| LDL-c |  |  |  |
| G1 | 1.00 (Ref) | 1.00 (Ref) | 1.00 (Ref) |
| G2 | 7.499 (2.022, 27.814)* | 1.321 (0.848, 2.058) | 2.874 (1.034, 7.986)* |
| G3 | 1.112 (0.084, 14.720) | 0.946 (0.492, 1.818) | 3.107 (1.184, 8.153)* |
| G4 | 3.477 (0.861, 14.042) | 0.534 (0.247, 1.153) | 0.222 (0.025, 1.982) |

G1 denoted low-to-low group, G2 denoted high-to-high group, G3 denoted high-to-low group, and G4 denoted low-to-high group, respectively; NA, not available due to insufficient number in the outcome group; **^#^** Adjusted for Maternal age, BMI, Conception by IVF, Family history of diabetes mellitus, Smoking and Multiparous; * *P* < 0.05.

**sTable 4 Association of trends in TG with trends in insulin resistance calculated by HOMA-IR in different BMI subgroups**

|  |  | **IR-H2** | **IR-H3** | **IR-H4** |
| --- | --- | --- | --- | --- |
| **BMI subgroups** | **Number (%)** | **aOR (95% CI) ^#^** | **aOR (95% CI) ^#^** | **aOR (95% CI) ^#^** |
| Underweight | 175 (14.2) |  |  |  |
| G1 | 142 (11.5) | 1.00 (Ref) | 1.00 (Ref) | 1.00 (Ref) |
| G2 | 7 (0.5) | NA | NA | 3.693 (0.329, 41.490) |
| G3 | 13 (1.1) | 2.846 (0.147, 55.157) | 2.873 (0.478, 17.275) | 2.402 (0.229, 25.187) |
| G4 | 13 (1.1) | NA | 1.069 (0.102, 11.185) | 21.871 (4.230, 113.098)* |
| Normal weight | 888 (72.0) |  |  |  |
| G1 | 597 (48.4) | 1.00 (Ref) | 1.00 (Ref) | 1.00 (Ref) |
| G2 | 133 (10.8) | 2.470 (1.391, 4.383)* | 1.605 (0.944, 2.728) | 2.389 (1.437, 3.969)* |
| G3 | 72 (5.8) | 1.995 (0.933, 4.265) | 0.763 (0.330, 1.761) | 1.248 (0.584, 2.668) |
| G4 | 86 (7.0) | 2.076 (1.020, 4.226)* | 1.211 (0.604, 2.429) | 2.241 (1.211, 4.146)* |
| Overweight or obesity | 171 (13.8) |  |  |  |
| G1 | 74 (6.0) | 1.00 (Ref) | 1.00 (Ref) | 1.00 (Ref) |
| G2 | 54 (4.4) | 1.925 (0.897, 4.131) | 1.559 (0.594, 4.092) | 0.359 (0.105, 1.227) |
| G3 | 28 (2.2) | 2.333 (0.901, 6.043) | 0.629 (0.146, 2.705) | 0.172 (0.021, 1.431) |
| G4 | 15 (1.2) | 0.862 (0.242, 3.065) | 1.705 (0.400, 7.269) | 1.388 (0.370, 5.209) |

G1 denoted low-to-low group, G2 denoted high-to-high group, G3 denoted high-to-low group, and G4 denoted low-to-high group, respectively; NA, not available due to insufficient number in the outcome group; **^#^** Adjusted for Maternal age, BMI, Conception by IVF, Family history of diabetes mellitus, Smoking and Multiparous; * *P* < 0.05.

**sTable 5 Association of trends in TG with trends in insulin resistance calculated by QUICKI in different BMI subgroups**

|  |  | **IR-Q2** | **IR-Q3** | **IR-Q4** |
| --- | --- | --- | --- | --- |
| **BMI subgroups** | **Number (%)** | **aOR (95% CI) ^#^** | **aOR (95% CI) ^#^** | **aOR (95% CI) ^#^** |
| Underweight | 175 (14.2) |  |  |  |
| G1 | 142 (11.5) | 1.00 (Ref) | 1.00 (Ref) | 1.00 (Ref) |
| G2 | 7 (0.5) | NA | NA | 3.693 (0.329, 41.490) |
| G3 | 13 (1.1) | 2.846 (0.147, 55.157) | 2.873 (0.478, 17.275) | 2.402 (0.229, 25.187) |
| G4 | 13 (1.1) | NA | 1.069 (0.102, 11.185) | 21.871 (4.230, 113.098)* |
| Normal weight | 888 (72.0) |  |  |  |
| G1 | 597 (48.4) | 1.00 (Ref) | 1.00 (Ref) | 1.00 (Ref) |
| G2 | 133 (10.8) | 2.487 (1.398, 4.422)* | 1.629 (0.956, 2.776) | 2.382 (1.436, 3.950)* |
| G3 | 72 (5.8) | 1.554 (0.683, 3.536) | 0.788 (0.340, 1.823) | 1.728 (0.871, 3.430) |
| G4 | 86 (7.0) | 1.949 (0.938, 4.052) | 0.994 (0.469, 2.108) | 2.341 (1.281, 4.277)* |
| Overweight or obesity | 171 (13.8) |  |  |  |
| G1 | 74 (6.0) | 1.00 (Ref) | 1.00 (Ref) | 1.00 (Ref) |
| G2 | 54 (4.4) | 1.925 (0.897, 4.131) | 2.030 (0.733, 5.620) | 0.359 (0.105, 1.227) |
| G3 | 28 (2.2) | 2.333 (0.901, 6.043) | 0.811 (0.181, 3.635) | 0.172 (0.021, 1.431) |
| G4 | 15 (1.2) | 0.862 (0.242, 3.065) | 2.227 (0.500, 9.910) | 1.388 (0.370, 5.209) |

G1 denoted low-to-low group, G2 denoted high-to-high group, G3 denoted high-to-low group, and G4 denoted low-to-high group, respectively; NA, not available due to insufficient number in the outcome group; **^#^** Adjusted for Maternal age, BMI, Conception by IVF, Family history of diabetes mellitus, Smoking and Multiparous; * *P* < 0.05.

# Supplementary Figures


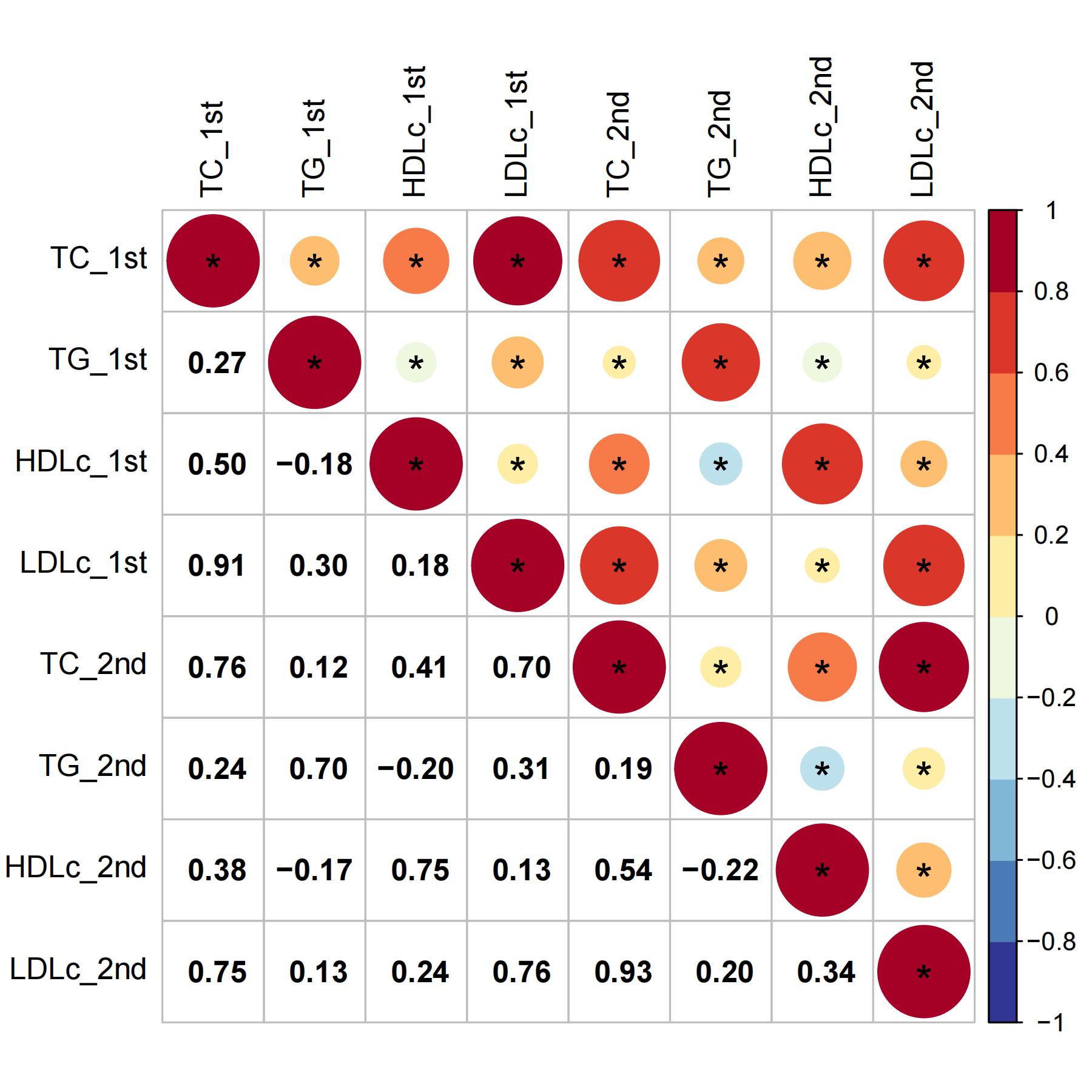


**sFigure 1. Correlation between lipid profiles in first and second trimesters**
